# Predicting the presence of coronary plaques featuring high-risk characteristics using polygenic risk scores and targeted proteomics in patients with suspected coronary artery disease

**DOI:** 10.1186/s13073-024-01313-8

**Published:** 2024-03-20

**Authors:** Peter Loof Møller, Palle Duun Rohde, Jonathan Nørtoft Dahl, Laust Dupont Rasmussen, Louise Nissen, Samuel Emil Schmidt, Victoria McGilligan, Daniel F. Gudbjartsson, Kari Stefansson, Hilma Holm, Jacob Fog Bentzon, Morten Bøttcher, Simon Winther, Mette Nyegaard

**Affiliations:** 1 Department of Biomedicine, Aarhus University, Aarhus, Denmark; 2 Department of Health Science and Technology, Aalborg University, Aalborg, Denmark; 3 Department of Cardiology, Gødstrup Hospital, Herning, Denmark; 4 Department of Clinical Medicine, Aarhus University, Aarhus, Denmark; 5 Personalized Medicine Centre, School of Medicine, Ulster University, Derry, Northern Ireland; 6 deCODE Genetics/Amgen, Inc, Reykjavik, Iceland; 7 School of Engineering and Natural Sciences, University of Iceland, Reykjavik, Iceland; 8 Faculty of Medicine, University of Iceland, Reykjavik, Iceland; 9 Centro Nacional de Investigaciones Cardiovasculares, Madrid, Spain; 10 Department of Cardiology, Aalborg University Hospital, Aalborg, Denmark

**Keywords:** High-risk coronary plaque, Coronary artery disease, Prediction, Genetics, Olink proteomics

## Abstract

**Background:**

The presence of coronary plaques with high-risk characteristics is strongly associated with adverse cardiac events beyond the identification of coronary stenosis. Testing by coronary computed tomography angiography (CCTA) enables the identification of high-risk plaques (HRP). Referral for CCTA is presently based on pre-test probability estimates including clinical risk factors (CRFs); however, proteomics and/or genetic information could potentially improve patient selection for CCTA and, hence, identification of HRP. We aimed to (1) identify proteomic and genetic features associated with HRP presence and (2) investigate the effect of combining CRFs, proteomics, and genetics to predict HRP presence.

**Methods:**

Consecutive chest pain patients (*n* = 1462) undergoing CCTA to diagnose obstructive coronary artery disease (CAD) were included. Coronary plaques were assessed using a semi-automatic plaque analysis tool. Measurements of 368 circulating proteins were obtained with targeted Olink panels, and DNA genotyping was performed in all patients. Imputed genetic variants were used to compute a multi-trait multi-ancestry genome-wide polygenic score (GPS_Mult_). HRP presence was defined as plaques with two or more high-risk characteristics (low attenuation, spotty calcification, positive remodeling, and napkin ring sign). Prediction of HRP presence was performed using the glmnet algorithm with repeated fivefold cross-validation, using CRFs, proteomics, and GPS_Mult_ as input features.

**Results:**

HRPs were detected in 165 (11%) patients, and 15 input features were associated with HRP presence. Prediction of HRP presence based on CRFs yielded a mean area under the receiver operating curve (AUC) ± standard error of 73.2 ± 0.1, versus 69.0 ± 0.1 for proteomics and 60.1 ± 0.1 for GPS_Mult_. Combining CRFs with GPS_Mult_ increased prediction accuracy (AUC 74.8 ± 0.1 (*P* = 0.004)), while the inclusion of proteomics provided no significant improvement to either the CRF (AUC 73.2 ± 0.1, *P* = 1.00) or the CRF + GPS_Mult_ (AUC 74.6 ± 0.1, *P* = 1.00) models, respectively.

**Conclusions:**

In patients with suspected CAD, incorporating genetic data with either clinical or proteomic data improves the prediction of high-risk plaque presence.

**Trial registration:**

https://clinicaltrials.gov/ct2/show/NCT02264717 (September 2014).

**Supplementary Information:**

The online version contains supplementary material available at 10.1186/s13073-024-01313-8.

## Background

Coronary computed tomography angiography (CCTA) is guideline-endorsed for identifying patients with suspected obstructive coronary artery disease (CAD) [1, 2]. Additionally, CCTA enables risk stratification including detection of high-risk plaques (HRP) predisposing patients to adverse cardiac events [3–5]. HRPs are defined as having two or more characteristics on the CCTA associated with a higher likelihood of having an acute coronary syndrome (ACS) [6]. These characteristics include low attenuation plaque, spotty calcification, positive remodeling, and the napkin ring sign.

In patients with stable chest pain suggestive of obstructive CAD, pre-test probability (PTP) estimation is recommended to guide decisions about downstream testing [1, 2]. Classically, PTP estimation is based on sex, age, and chest pain typicality [1]. In general, both patient stratification, diagnostic value, and risk prediction have been improved by also considering clinical risk factors (CRFs) [7, 8] in the PTP estimation. The majority of de novo chest pain patients have low PTP and are recommended to undergo index testing by CCTA to identify potentially obstructive lesions [1, 2, 7]. Evidence of HRP characteristics has been documented in both patients with obstructive and non-obstructive lesions which CCTA outlines [4, 9], and as early treatment of patients with HRP characteristics could improve prognosis [10], an optimized clinical tool to guide CCTA utilization for HRP identification is warranted.

Based on large-scale proteomic panels and genotyping arrays yielding multi-protein models and genome-wide polygenic scores (GPSs), respectively, proteomics and genetics have been used for the prediction of obstructive CAD [11, 12], CAD-related traits [13], and general CAD risk management [14]. However, whether the combination of proteomic and genetic data improves the prediction accuracy of HRP presence is unknown. Further, a high PRS of CAD has previously been shown to be most predictive in patients younger than 55 years of age [15]. It is currently unknown whether this is also the case for HRP presence.

The primary aim of this study was to identify features (i.e., CRF, GPS, and proteins) associated with HRP presence and evaluate a combination of clinical, proteomic, and genetic data to predict the trait in de novo chest pain patients with suspected obstructive CAD. Secondly, we aimed to uncover any age-related variation in predictive features.

## Methods

### Study population

This is a sub-study of the Danish study of Non-Invasive testing in Coronary Artery Disease (Dan-NICAD) 1 study. The study protocol and main results have previously been described [16, 17]. In short, Dan-NICAD 1 was a prospective, multicenter cross-sectional study of 1675 patients with no previous history of CAD, low-intermediate PTP, and symptoms suggestive of obstructive CAD referred for initial testing by CCTA. On the day of CCTA, patients also underwent blood sampling, while symptoms and cardiac risk factors were registered. EDTA plasma was isolated from blood samples and stored at -80 °C.

### CCTA add HRP definition

CCTA was performed on a 320-slice volume CT scanner (Aquilion One; Toshiba Medical Systems, Japan) following usual clinical guidelines. The presence and location of coronary plaques and CAD severity assessment were evaluated by a cardiologist [16]. CCTA plaque analysis was performed blinded to clinical risk factors, proteomics, and genetics, using the previously validated software Qangio CT (Research Edition ver. 3.1.4.2, Medis NL [18]).

Obstructive CAD was defined as a 50% diameter stenosis by visual assessment at CCTA. Plaques were evaluated by dedicated and trained core laboratory personnel (Cardiac Imaging Center, Department of Cardiology, Goedstrup Hospital, Denmark) for four qualitative high-risk characteristics: (1) positive remodeling (remodeling index > 1.10 calculated as vessel area at the location of plaque divided by reference vessel area in adjacent normal segments), (2) low-attenuation (non-calcified plaque with a plaque volume of > 1 mm [3] containing voxels with CT attenuation < 30 Hounsfield Units), (3) spotty calcification (< 3 mm calcium encased in non-calcified plaque), and (4) napkin ring sign (ring-like structure with lowest CT attenuation in the center of the plaque). The presence of HRP was defined as having coronary lesions with two or more high-risk characteristics, as recommended by the CAD-RADS 2.0 system [19].

### Clinical risk factor model

The clinical risk factor model was based on the features incorporated in the risk factor-weighted clinical likelihood (RF-CL) model of obstructive CAD [7]. The model is based on sex, age, angina typicality (typical, atypical, non-specific, and dyspnea), and the number of risk factors (family history of early CAD, smoking [never vs. active/former], dyslipidemia [receiving cholesterol-lowering medication], hypertension [receiving antihypertensive medication], and type 2 diabetes) ranging from 0 to 5. Models utilizing CRFs included all of the above features in their input.

### Protein model

In total, 368 proteins were measured in EDTA plasma samples at Olink proteomics AB (Uppsala, Sweden) as previously described [20]. The protein analysis was done using four Olink Target 96 panels: the Cardiovascular II, Cardiovascular III, Inflammation, and Immune Response panel. The Cardiovascular II and III panels were chosen for their relevance to cardiovascular processes, while the Inflammation and Immune Response panels were included to broaden the search for associations. Samples marked in Olink’s internal quality control steps were removed. Also, if the fraction of samples below the limit of detection (LOD) for a protein exceeded 20%, the protein was excluded from further analyses. For all included proteins, values were rank-normalized before adjusting for the collection box. A total of 300 proteins passed quality control and were included in the input to all models utilizing proteomic features.

### Genetic model

Genotyping, quality control, and imputation have previously been described [20]. In short, genotyping used the Illumina Global Screening Array, followed by imputation using the Michigan Imputation Server. Single nucleotide polymorphisms (SNPs) with minor allele frequencies ≤ 1% were excluded.

The genotype weights of the multi-trait multi-ancestry genome-wide polygenic score (GPS_Mult_, https://www.pgscatalog.org/score/PGS003725/) by Patel et al. [21] were used to estimate genetic risk for CAD, as no dedicated HRP polygenic score exists. GPS_Mult_ aggregates multiple polygenic scores from CAD, BMI, ischemic stroke, diabetes mellitus, peripheral artery disease, glomerular filtration rate, systolic and diastolic blood pressure, LDL and HDL cholesterol, and triglycerides utilizing the 1,296,172 variants included in HapMap3.

### Combined models

For combined models, the prediction was based on three groups of input features: (1) clinical risk factors (nine features), (2) proteins (300 features), and (3) GPS_Mult_ (one feature), with the name of each model reflecting the included feature groups.

### Data analysis

The primary outcome was defined as the presence of HRP. For individual features (i.e., individual clinical risk factors, individual proteins, and GPS_Mult_), performance was measured using the receiver operating characteristic (ROC) with the area under the curve (AUC) and 95% confidence interval (CI) implemented in the pROC [22] R package. Significance testing was performed using the Wilcoxon rank sum test and a Bonferroni corrected significance threshold of 0.05/315.

Models using more than one input feature were constructed using logistic regression with elastic net regularization [23, 24] implemented in the glmnet and caret [25] R packages, using weights to compensate for case–control imbalance. To limit the impact of random sampling noise, all models were based on 100 repeats of fivefold cross-validation (CV), foregoing a completely independent test set. ROC was used as a summary metric to select the optimal model. Hyperparameters were chosen from a combination of three alphas (0.1, 0.55, 1) and 10 lambdas ranging from 0.0001 to 1.

For all models, performance in every fold and repeat was stored, resulting in 500 AUC estimates for each model. Performance was reported as the mean ± standard error of all AUC estimates. As the AUC distributions for some models were not Gaussian, comparisons of model performance used the Kruskal–Wallis rank sum test to compare all models followed by Bonferroni corrected Dunn’s test for individual post-hoc comparisons, reporting the adjusted *P* value.

Estimation of predictive performance in plaque subtypes was performed for all models. For each model, the HRP probability was averaged per individual across the 100 repeats, before calculation of the AUC with 95% CI for single features.

All analyses were performed within R [26] version 4.2.1. SHapley Additive explanation (SHAP) values were estimated using the fastshap [27] R package, using 100 Monte Carlo repetitions. Plotting was performed using ggplot2 [28].

## Results

### Study population

Of the 1675 eligible patients, 1462 (87%) had complete data on clinical risk factors, proteomics, genomics, and CCTA. Baseline demographics are shown in Table 1. Baseline demographics of excluded patients are shown in Table S1, while the reason for exclusion is shown in Fig S1.

**Table 1:** Baseline information

	**Overall** (*n* = 1462)		**High-risk plaque** (*n* = 165)		**No high-risk plaque** (*n* = 1297)	
**Demographics**						
Age, years	57 ± 9		60 ± 8		57 ± 9	
Males	699 (48%)		122 (74%)		577 (44%)	
***Risk factors, n (%)***						
Family history	527 (36%)		63 (38%)		464 (36%)	
Current/former smoker	778 (53%)		104 (63%)		674 (52%)	
Dyslipidemia	349 (24%)		42 (25%)		307 (24%)	
Hypertension	517 (35%)		69 (42%)		448 (35%)	
Type 2 diabetes	85 (6%)		16 (10%)		69 (5%)	
***Type of chest pain, n (%)***						
Typical angina	393 (27%)		63 (38%)		330 (25%)	
Atypical angina	497 (34%)		47 (28%)		450 (35%)	
Non-specific chest discomfort	271 (19%)		23 (14%)		248 (19%)	
Dyspnea	301 (21%)		32 (19%)		269 (21%)	
***Laboratory tests***						
Cholesterol medication	349 (24%)		42 (25%)		307 (24%)	
	Yes	No	Yes	No	Yes	No
Total cholesterol, mmol/L^a^	4.9 ± 1.2	5.5 ± 1.0	5.2 ± 1.6	5.8 ± 1.4	4.9 ± 1.1	5.5 ± 1.0
LDL cholesterol, mmol/L^a^	2.8 ± 1.1	3.4 ± 0.9	3.1 ± 1.2	3.7 ± 0.9	2.8 ± 1.0	3.4 ± 0.9
HDL cholesterol, mmol/L^a^	1.4 ± 0.4	1.5 ± 0.5	1.4 ± 0.4	1.4 ± 0.5	1.5 ± 0.4	1.5 ± 0.4
Triglyceride, mmol/L^a^	1.5 [1.0–2.1]	1.3 [0.9–1.9]	1.6 [1.0–2.3]	1.5 [1.1–2.3]	1.5 [1.0–2.1]	1.3 [0.9–1.9]
***Measurements***						
Blood pressure medication	517 (35%)		69 (42%)		448 (35%)	
	Yes	No	Yes	No	Yes	No
Systolic blood pressure, mm Hg^a^	143 ± 19	136 ± 18	144 ± 18	146 ± 19	143 ± 19	135 ± 18
Diastolic blood pressure, mm Hg^a^	84 ± 11	82 ± 11	83 ± 11	86 ± 11	85 ± 11	81 ± 11
Body mass index^a^	26.8 ± 4.2		26.3 ± 3.6		26.8 ± 4.3	
Obstructive CAD at CCTA	341 (23%)		134 (81%)		207 (16%)	
Coronary artery calcium score^a^	0 [0–81]		210 [52–624]		0 [0–42]	

*Abbreviations*: *CAD* coronary artery disease, *CCTA* coronary computed tomography angiography, *LDL* low-density lipoprotein, *HDL* high-density lipoprotein

Values are listed as mean ± standard deviation for normally distributed data; otherwise, the median and interquartile range are used

^a^Missing values were observed in total cholesterol *n* = 41, LDL cholesterol *n* = 41, HDL cholesterol *n* = 38, triglyceride *n* = 46, systolic and diastolic blood pressures *n* = 3, body mass index *n* = 8, coronary artery calcium score *n* = 1

**Fig. 1:**
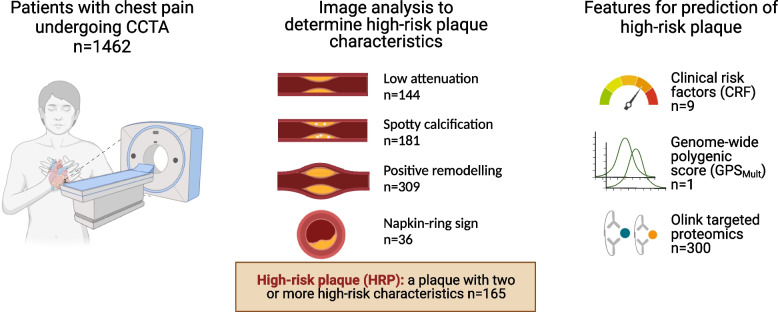
Study design. 1462 patients underwent coronary computed tomography angiography (CCTA), followed by image analysis of high-risk plaque (HRP) characteristics. Finally, nine clinical risk factors, one multi-trait multi-ancestry genome-wide polygenic score (GPS_Mult_), and 300 proteins were used to predict HRP presence

High-risk characteristics including positive remodeling, low-attenuation, spotty calcification, and napkin-ring sign were present in 309 (21%), 144 (10%), 181 (12%), and 36 (2%) patients, respectively (Fig. 1). In total, HRP presence was identified in 165/1462 (11%) patients, and 341/1462 patients (23%) had obstructive CAD. HRP presence was significantly correlated with obstructive CAD presence (Pearson’s correlation = 0.49, *P* < 0.001), with 134 patients having both.

### Single clinical, protein, and genetic feature association with high-risk plaque

**Fig. 2:**
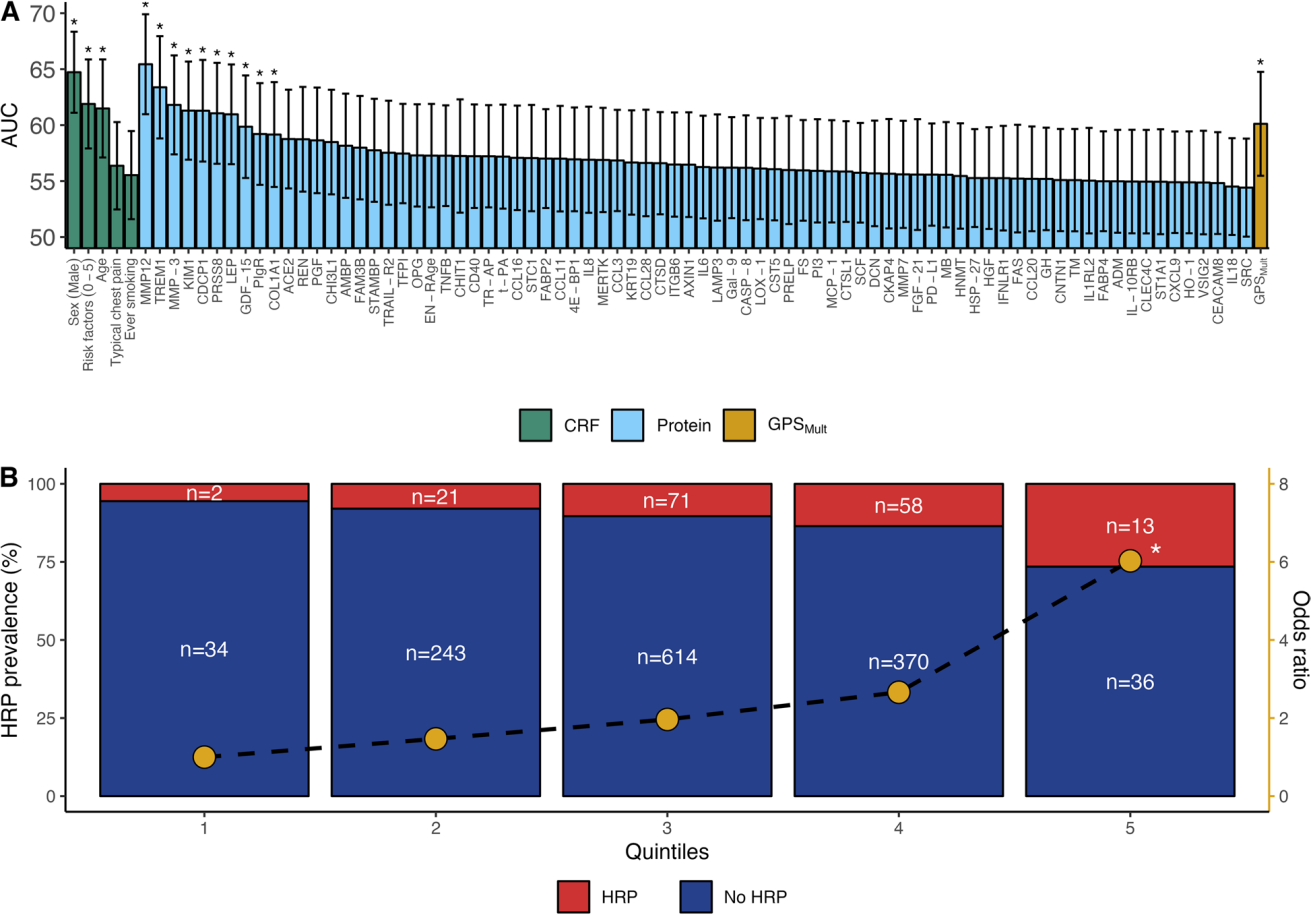
Predictive performance of single features. **A** Area under the curve (AUC) for individual features, grouped by feature type. Error bars indicate a 95% confidence interval (CI). Asterisks indicate statistical significance after Bonferroni correction for multiple testing. CRF, clinical risk factors; GPS_mult_, multi-trait multi-ancestry genome-wide polygenic score. Only single features with a lower limit of 95% CI above 50% are shown.** B** Prevalence of high-risk plaque (HRP) stratified by GPS_Mult_ quintiles. Odds ratios are calculated using the first quintile as a reference. Asterisk indicates a statistically significant difference in the estimated odds ratio compared to the reference quintile

A total of 84 individual clinical, protein, and genetic features had a 95% CI lower limit of the AUC estimate above 50% (Fig. 2A). Among those, 14/84 (17%) features were significantly associated with HRP presence when accounting for multiple testing: three clinical risk factors (age, sex (male) and number of risk factors (0–5)), ten proteins (MMP12, TREM1, MMP-3, KIM1, CDCP1, PRSS8, LEP, GDF-15, PIgR, and COL1A1), and GPS_Mult_. Stratifying patients by GPS_Mult_ quintiles revealed increased HRP prevalence with increasing polygenic score, with the highest quintile having an odds ratio of 6.03 (*P* = 0.02) relative to the lowest quintile (Fig. 2B).

### Combined prediction models of high-risk plaque

**Fig. 3:**
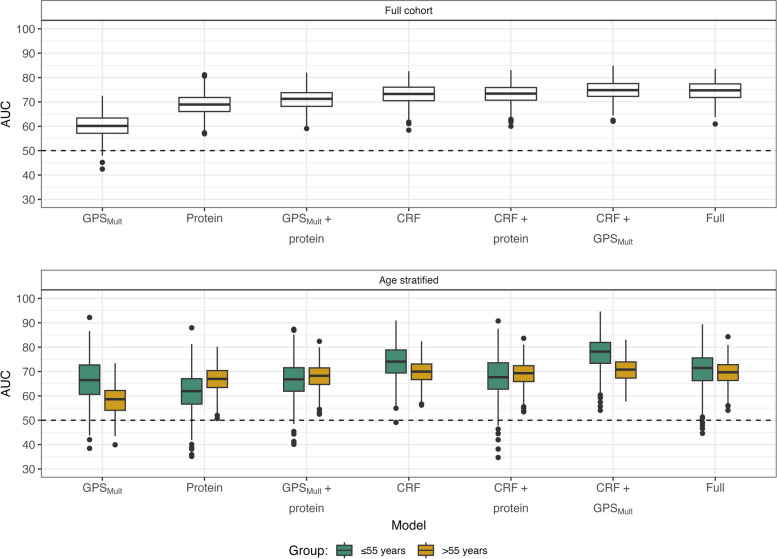
Predictive performance of individual and combined models. Models included features from up to three feature groups, as shown on the *x*-axis, revealing the CRF + GPS_Mult_ model to be the most predictive in the full cohort. Stratifying patients by age group resulted in improved GPS_Mult_ performance in the group ≤ 55 years of age, while the CRF + GPS_Mult_ models were best in both groups. AUC, area under the curve; CRF, clinical risk factor; GPS_Mult_, multi-trait multi-ancestry genome-wide polygenic score; Full, CRF + protein + GPS_Mult_

Compared to the model based on CRF features, the prediction accuracy of HRP presence by the protein model was lower (AUC 73.2 ± 0.1 vs. 69.0 ± 0.1, *P* < 0.001) (Fig. 3A). The GPS_Mult_ model had an AUC of 60.1 ± 0.1, which was inferior to both the CRF and protein models.

Combining GPS_Mult_ with CRFs or protein features increased prediction accuracy of HRP presence (CRF + GPS_Mult_: 74.8 ± 0.1, *P* = 0.004; Protein + GPS_Mult_ = 71.0 ± 0.1, *P* < 0.001). Combining CRF and protein features leads to the inclusion of eight features in total, four of these being proteins, but no improvement in the accuracy of prediction was observed compared to the CRF model (73.2 ± 0.1, *P* = 1.00). Similarly, a full model including all feature groups did not improve the CRF + GPS_Mult_ model (74.6 ± 0.1, *P* = 1.00).

Across all models, using each model for predicting individual high-risk plaque characteristics showed the lowest predictive performance for low attenuation plaques and the highest for napkin ring sign (Table 2).

**Table 2:** Plaque subtype prediction

**Models**	**Low attenuation** (*n* = 144)	**Spotty calcification** (*n* = 181)	**Positive remodeling** (*n* = 309)	**Napkin ring sign** (*n* = 36)
CRF	71.1 [66.8–75.4]	72.7 [69.0–76.3]	71.9 [68.7–75.0]	76.9 [69.4–84.3]
Protein	67.4 [62.9–71.8]	69.3 [65.3–73.4]	69.0 [65.6–72.3]	74.2 [66.5–81.9]
GPS_Mult_	59.2 [54.3–64.1]	60.2 [55.9–64.5]	62.7 [59.2–66.1]	61.1 [51.6–70.6]
CRF + GPS_Mult_	72.8 [68.6–77.0]	75.1 [71.7–78.5]	74.9 [71.9–77.8]	78.0 [70.0–85.9]
Protein + GPS_Mult_	69.1 [64.9–73.3]	71.5 [67.7–75.3]	71.9 [68.8–75.0]	76.3 [69.3–83.3]
CRF + Protein	71.1 [66.6–75.6]	73.0 [69.3–76.8]	71.5 [68.3–74.7]	79.3 [72.7–85.9]
Full	72.3 [68.3–76.4]	75.0 [71.5–78.6]	74.9 [72.0–77.9]	79.5 [72.8–86.2]

*CRF* clinical risk factor, *GPS*_*Mult*_ multi-trait multi-ancestry genome-wide polygenic score

Numbers are the area under the curve with a 95% confidence interval

Supplementary analysis defining HRP as 1- or 3-feature positive plaques (instead of 2-feature) found CRF to have the best predictive ability in 3-feature HRP (*n* = 29), while CRF + GPS_Mult_ had the best predictive ability for 1-feature HRP (*n* = 388, Table S2).

### Age stratification

Stratifying the cohort into patients above and below 55 years of age showed an HRP prevalence of 8.0% (50/627) in patients ≤ 55 years and 13.8% (115/835) in patients > 55 years. For all non-protein models, this stratification improved HRP prediction in patients ≤ 55 years (Fig. 3) relative to patients > 55 years. Especially the GPS_Mult_ among patients ≤ 55 displayed improved prediction accuracy significantly (AUC_≤55_ = 66.5 ± 0.4 vs. AUC_>55_: 58.3 ± 0.2, *P* < 0.001). The best-performing model in both age groups was based on CRF and GPS_Mult_, yielding improved prediction in younger patients (AUC_≤55_ = 77.3 ± 0.3 vs. AUC_>55_ = 70.7 ± 0.02 (*P* < 0.001)).

Predicting individual high-risk plaque characteristics in patients ≤ 55 years of age showed similar results as in the full cohort (Table S3).

**Fig. 4:**
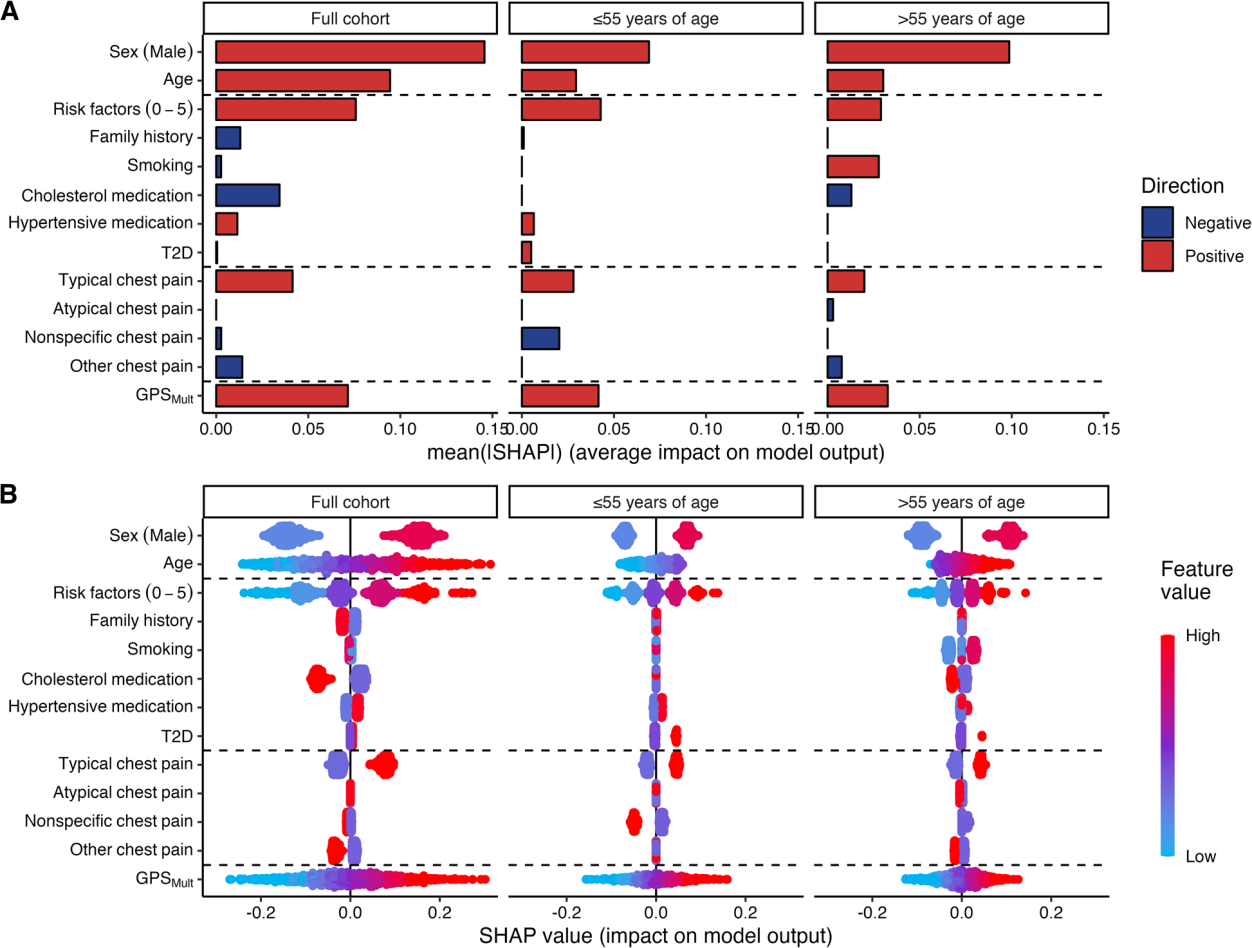
Feature importance of the CRF + GPS_Mult_ models across cohort groups.** A** Mean absolute SHAP values, representing the average importance of input features on model prediction, with blue features leading to lower risk and red features leading to higher risk. **B** Patient-level SHAP values, showing how impactful some features can be in extreme cases. SHAP, Shapley additive explanation; T2D, type 2 diabetes mellitus; GPS_Mult_, multi-trait multi-ancestry genome-wide polygenic score

Comparing the impact of individual features (utilizing SHAP values) on model output in the CRF + GPS_Mult_ models across the three groups (full cohort, ≤ 55 years of age, and > 55 years of age), sex was consistently the most influential single feature on HRP presence (Fig. 4A). Comparing the impact of CFRs to GPS_Mult_ revealed GPS_Mult_ to be equally important to the sum of risk factors. For individual risk factors, family history of early CAD appeared protective in the full cohort, smoking had an impact in patients > 55 years of age, and cholesterol medication was protective in patients > 55 years of age and the full cohort (Fig. 4A). Finally, the use of anti-hypertensive medication and having diabetes both had a low impact on all model predictions. Typical chest pain symptoms indicated increased HRP risk in all groups, while nonspecific chest pain in patients ≤ 55 years of age and other chest pain in patients > 55 years of age and the full cohort indicated reduced HRP risk.

Examining the individual SHAP values (Fig. 4B) revealed multiple features (age, number of risk factors, and GPS_Mult_) with the potential to be more impactful than sex for a single patient although they were inferior from a cohort perspective.

## Discussion

The main finding of the study was that HRP presence can be predicted using clinical risk factors, whereas genetic data primarily serves as a strong source of complementary information, resulting in improved HRP prediction in the complete cohort and especially among patients below 55 years of age the prediction accuracy was significantly improved. Finally, we did not find that our proteomic data improved the overall discrimination, despite having better individual prediction accuracy than genetic data.

Looking at individual input features, 14 features were significantly associated with HRP presence, with well-known clinical risk factors and proteins showing superior discrimination. Our findings are not surprising as several proteins are correlated with sex and age which independently correlates to HRP presence (Fig. 2A). In our study, we used protein measurements without adjusting the protein level for age and sex, as we wanted to retain these signals for models which did not incorporate age and sex directly.

Regarding the combined models, CRF features were the most predictive feature group for HRP presence. In contrast, Bom et al. [13] reported 196 patients with 22% HRP prevalence and utilized four Olink panels, three of which were also analyzed in our study, and found proteins to be predictive of HRP presence (AUC of 79 ± 1) with impaired prediction accuracy by CRFs alone (AUC of 65 ± 4). The difference in CRF predictive performance between our studies could be explained by our study having more detailed CRF data, e.g., the number of risk factors as implemented in the RF-CL model. Furthermore, the higher predictive performance of proteomics observed by Bom et al. may be explained by their inclusion of the targeted Cardiometabolic Olink panel which was not included in our study. This highlights the possibility of future discoveries as proteomic panels include an increasing number of proteins.

Due to the rarity of cohorts with detailed high-risk plaque characteristics, no suitable GWAS summary statistic exists for the calculation of a traditional polygenic score for HRP. Instead, we leveraged GPS_Mult_, an existing polygenic score for CAD, which incorporates genetic information about multiple known CAD risk factors and from multiple ancestries. GPS_Mult_ showed the greatest prediction accuracy in patients below 55 years of age. This is consistent with previous findings by Mars et al. reporting PRS for CAD prediction to have lower performance in people above 55 years of age [15] and also explains the somewhat low predictive ability of GPS_Mult_ by itself in our study (mean age of 60 years).

Interestingly, we found that GPS_Mult_ was able to improve prediction when combined with either CRFs or proteins, indicating that genetics carry additional information about existing or novel aspects of HRP risk. Meanwhile, the inclusion of proteins with CRFs did not improve prediction, suggesting that the proteins assessed in our study do not contain additional information about HRP risk. It is possible that future improvements in HRP prediction could arise from increasing the number of included plasma proteins, using, e.g., SomaScan 7K or Olink Explore HT.

One of the strengths of this study is the consecutive enrollment of nearly 1500 patients with detailed CCTA readings allowing for the discovery of HRP presence and specific plaque characteristics. Additionally, the Danish healthcare system is likely to reduce referral bias, as no direct payment is needed for citizens to undergo diagnostic testing and therefore our findings represent an all-comer population.

The primary limitation of the study is the lack of external validation. Instead, this study utilized internal testing through repeated cross-validation, enabling visualization of the uncertainty of our performance estimates (Fig. 3). Additionally, this study investigated only 300 proteins, meaning that additional studies, using larger, more explorative panels, are required to investigate the remaining plasma proteome.

## Conclusion

Genetic data can be used to improve both clinical risk factors and proteomic models for the prediction of HRP presence, especially in patients below 55 years of age. However, a model combining both clinical risk factors and proteomics did not improve high-risk plaque identification, despite both types of data being predictive on their own.

### Supplementary Information

**Additional file 1: Table S1.** Baseline information. **Table S2.** Analysis of 1, 2, and 3 feature plaques. **Table S3.** Plaque subtype prediction in patients ≤55 years of age. **Fig S1.** Patient inclusion.

**Additional file 2: Table S4.** Summary level data of number of risk factors, proteomics and GPS_Mult_ stratified by case/control status.

## Data Availability

Summary level data of number of risk factors, proteomics, and GPS_Mult_ are available in Table S4. Individual level data cannot be made publicly available.
