# Supplementary material for: Predicting the presence of coronary plaques featuring high-risk characteristics using polygenic risk scores and targeted proteomics in patients with suspected coronary artery disease
Source: Genome Med. 2024 Mar 20;16:40. doi: 10.1186/s13073-024-01313-8 (PMC10953133; doi:10.1186/s13073-024-01313-8)
Supplement: Supplementary file 1 — Additional file 1: Table S1. Baseline information. Table S2. Analysis of 1, 2, and 3 feature plaques. Table S3. Plaque subtype prediction in patients ≤55 years of age. Fig S1. Patient inclusion. [file 13073_2024_1313_MOESM1_ESM.pdf]

Tables

Table S1 – Baseline information

**Demographics**	**Included** (n = 1462)		**Excluded** (n = 213)	
Age, years	57 ± 9		57 ± 9	
Males	699 (48%)		105 (49%)	
Family history [a]	527 (36%)		89 (43%)	
Current/former smoker [a]	778 (53%)		35 (17%)	
Dyslipidemia [a]	349 (24%)		40 (22%)	
Hypertension [a]	517 (35%)		63 (32%)	
Type 2 diabetes [a]	85 (6%)		9 (4%)	
Type of chest pain, n (%) [a]				
Typical angina	393 (27%)		63 (30%)	
Atypical angina	497 (34%)		65 (31%)	
Non-specific chest discomfort	271 (19%)		29 (14%)	
Dyspnea	301 (21%)		45 (21%)	
Cholesterol medication [a]	349 (24%)		40 (22%)	
	Yes	No	Yes	No
Total cholesterol, mmol/L [a]	4.9±1.2	5.5±1.0	5.0±1.2	5.5±1.0
LDL cholesterol, mmol/L [a]	2.8±1.1	3.4±0.9	2.8±1.1	3.5±0.9
HDL cholesterol, mmol/L [a]	1.4±0.4	1.5±0.5	1.5±0.6	1.4±0.4
Triglyceride, mmol/L [a]	1.5 [1.0-2.1]	1.3 [0.9-1.9]	1.5 [1.0-2.0]	1.3 [1.0-2.1]
Blood pressure medication [a]	517 (35%)		63 (32%)	
	Yes	No	Yes	No
Systolic blood pressure, mm Hg [a]	143±19	136±18	144±18	136±19
Diastolic blood pressure, mm Hg [a]	84±11	82±11	86±10	82±12
Body mass index [a]	26.8±4.2		27.1±4.6	
Obstructive CAD at CCTA [a]	341 (23%)		50 (23%)	
Coronary artery calcium score [a]	0 [0-81]		0 [0-95]	

Values are listed as mean ± standard deviation for normally distributed data, otherwise the median and interquartile range is used. [a] Missing values were observed in: Family history n = 7, smoking status n = 8, dyslipidemia n = 30, hypertension n = 16, type 2

diabetes n = 7, type of chest pain n = 11, cholesterol medication n = 30, total cholesterol n = 8, LDL cholesterol n=8, HDL cholesterol n=8, triglyceride n=10, blood pressure medication n = 16, systolic and diastolic blood pressures n=1, body mass index n=1, obstructive CAD at CCTA n = 15, coronary artery calcium score n=11; Abbreviations: CAD, coronary artery disease; CCTA, coronary computed tomography angiography; LDL, low density lipoprotein; HDL, high density lipoprotein.

Table S2 – Analysis of 1, 2, and 3 feature plaques

Models	1-feature plaque (n = 388)	2-feature plaque (n = 165)	3-feature plaque (n = 29)
CRF	73.4 ± 0.1	73.2 ± 0.1	77.2 ± 0.2
Protein	69.9 ± 0.1	69.0 ± 0.1	60.7 ± 0.3
GPS_{Mult}	62.3 ± 0.1	60.1 ± 0.1	40.3 ± 0.2
CRF + GPS_{Mult}	76.4 ± 0.1	74.8 ± 0.1	76.3 ± 0.2
Protein + GPS_{Mult}	73.0 ± 0.1	71.0 ± 0.1	60.5 ± 0.3
CRF + Protein	73.8 ± 0.1	73.2 ± 0.1	70.8 ± 0.2
Full	76.4 ± 0.1	74.6 ± 0.1	70.8 ± 0.2

Numbers are area under the curve ± standard error. 2-feature plaque corresponds to high-risk plaque as defined in the main manuscript.

Table S3 – Plaque subtype prediction in patients ≤ 55 years of age

Models	Low attenuation (n = 52)	Spotty calcification (n = 49)	Positive remodeling (n = 83)	Napkin ring sign (n = 8)
CRF	69.5 [62.3-76.7]	75.9 [70.1-81.8]	69.2 [63.4-75.1]	73.8 [54.1-93.5]
Protein	58.4 [50.2-66.5]	59.9 [51.7-68.1]	59.3 [52.6-65.9]	68.7 [45.8-91.7]
GPS_{Mult}	63.3 [55.4-71.1]	67.9 [59.5-76.3]	67.5 [61.0-74.0]	60.0 [32.2-87.7]
CRF + GPS_{Mult}	72.1 [65.3-79.0]	79.4 [73.9-85.0]	73.8 [68.5-79.2]	76.8 [55.7-97.9]
Protein + GPS_{Mult}	61.8 [54.1-69.5]	65.3 [57.7-73.0]	63.9 [57.7-70.1]	67.6 [45.3-89.8]
CRF + Protein	61.7 [53.6-69.7]	73.0 [66.3-79.7]	64.1 [57.7-70.4]	79.1 [68.3-90.0]
Full	65.8 [58.6-73.0]	78.4 [72.1-84.6]	70.2 [64.6-75.8]	76.7 [55.0-98.4]

Numbers are area under the curve with 95% confidence intervals.

Figures

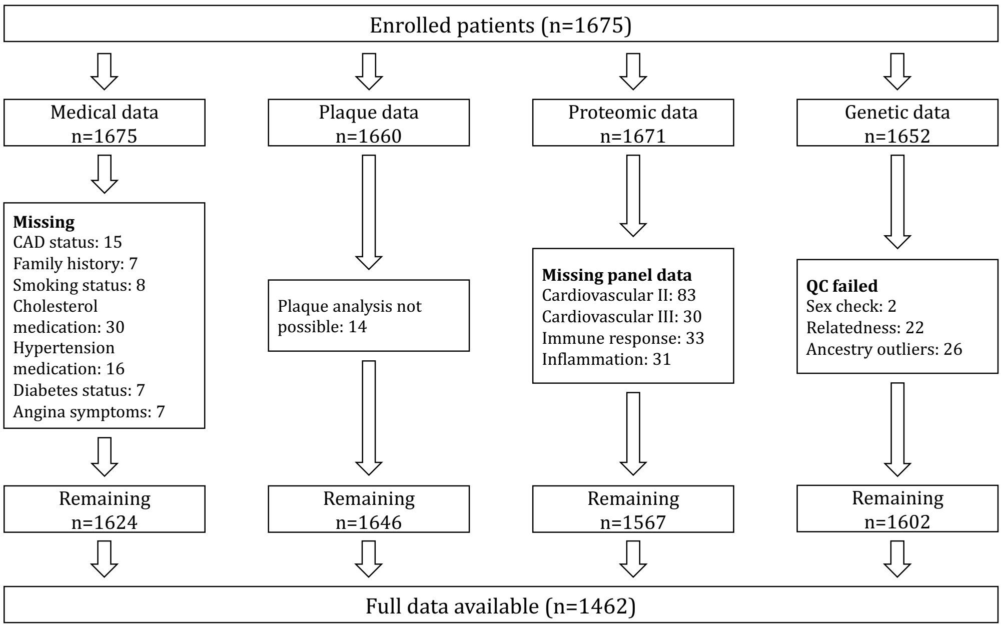

Fig S1. Patient inclusion. 1675 patients were enrolled, combining medical, plaque, proteomic and genetic data, resulting in 1462 patients with full data available.
